# Traditional sexing methods and external egg characteristics combination allow highly accurate early sex determination in an endangered native turkey breed

**DOI:** 10.3389/fvets.2022.948502

**Published:** 2022-08-15

**Authors:** J. I. Salgado Pardo, Francisco Javier Navas González, Antonio González Ariza, A. Arando Arbulu, J. M. León Jurado, J. V. Delgado Bermejo, M. E. Camacho Vallejo

**Affiliations:** ^1^Department of Genetics, Faculty of Veterinary Sciences, University of Córdoba, Córdoba, Spain; ^2^Department of Agriculture and Ecological Husbandry, Area of Agriculture and Environment, Andalusian Institute of Agricultural and Fisheries Research and Training (IFAPA), Córdoba, Spain; ^3^Agropecuary Provincial Centre, Córdoba Provincial Government, Córdoba, Spain

**Keywords:** external egg quality, poult morphological, sex determination, preincubation, post-hatching, native breed, behavior

## Abstract

Early sex determination methods are not only crucial in the worldwide massive poultry industry, but also for small-holder producers. The profitability of sexing techniques must be accounted for when aiming to boost management, nutrition, and conservation practices in endangered poultry breeds. This becomes pivotal when the local breed dealt with belongs to an understudied species, such as the turkey. So, the main objective of this study is to identify which method combination may report a higher likelihood of successful sex determination in poults across the three-pattern varieties of the Andalusian turkey breed. A total of 84 one to two days old Andalusian turkey poults (42 black, 28 black-roan, and 14 bronze-roan) were evaluated in this study. Sex determination was performed using 15 methods, which included testing external egg metrics and eggshell color, poult morphological appraisal and phaneroptics, and behavioral traits. Possible differences across plumage varieties and the interaction between sex and plumage were observed when external egg quality was measured. Sex determination through behavioral methods in black base feathered (black and black-roan) male sex individuals showed seven times higher sensitivity when compared to the rest of the studied individuals (χ^2^ = 7.14, df = 1, *P* < 0.01). In contrast, for the black-roan plumage females, the method based on the color of down feathers was approximately four times more sensitive (χ^2^ = 3.95, df = 1, *P* ≤ 0.05). For the bronze-roan pattern, none of the sexing techniques was reported to efficiently predict sex itself. However, the most proper method combination to determine sex, independent of plumage color, was physical external egg characteristics, the color of down feathers, and behavioral approaches (“English method” and “slap technique”). The specificity values were found to be 49.12, 93.33, and 100%, while the sensitivity values were observed to be 74.64, 91.03, and 100%, which translated into accuracy of 63.10, 92.26, and 100% in black, black-roan, and bronze-roan poults, respectively. Our results suggest that the method combination tested in this study could be considered a highly accurate, simple, and affordable alternative for sex determination in turkeys. This could mean a pivotal advance for small producers of turkeys, as early sex detection can help to plan timely conservational management strategies, which is of prominent importance in the context of endangered poultry breeds.

## Introduction

Early sex determination plays a pivotal role in the turkey farming specialization, since two different lines are commonly used: a heavy line, which comprises males, and a laying line, which sources dams (1). The difference in body weights between these two strains is the basis for the differentiation of farms to ensure basic animal management and nutrition (2). Thus, hatching poults need to be separated by sexes to be raised independently, depending on the commercial strategy chosen by breeders (3). Apart from its critical economic impact, the possibility of sex detection before hatching is also interesting in terms of both animal welfare and ethical issues by the early separation of the different sexes (4).

Sexing chicks during the first day of life could be a critical step not only in the commercial poultry industry but also in the design of conservational and breeding programs for endangered native breeds, as described by (5). The use of reliable sexing techniques in endangered avian breeds is of special importance in breeding programs, since it could avoid lowing hatching rate problems or copulation problems due to side effects derived from high inbreeding in such minority populations (6). In these terms, native poultry breeds, such as the Andalusian turkey, could benefit from the early sex determination of poults. The Andalusian turkey breed is a Spanish endangered autochthonous population distributed around the Southeast Iberian Peninsula and might be the direct descendants of the first turkeys imported from Mexico to arrive in Seville's port during the early 16th century (7).

Andalusian turkey is raised in semi-grazing conditions by backyard producers in the Guadalquivir Basin and is characterized by great adaptability to the environment. However, during the 20th century, the number of individuals belonging to this native breed drastically decreased as a consequence of the introduction of commercial hybrid strains in Spain (8). This situation promoted local genotype displacement and hybridization, which suggested the need for urgent conservation measures to be taken.

The implementation of a standardized accurate method for the sex determination of 1-day-old poults could mean a crucial improvement for breeders, making it possible to take proper management decisions at hatching instead of waiting for 4–5 months, when animals start to display sexual dimorphism characters (9). As a consequence, Andalusian turkey males may be aimed toward the maximization of their meat production while letting hen for the laying aptitude (10). These sexing methods can also be a beneficial tool when management strategies to preserve genetic diversity are designed, since sex distribution across the population is possibly biased (5).

Sexual dimorphism is defined as the differences in external appearance, among other traits, between the two genders of one species and is influenced by both genetic and environmental factors (11). Generally, males and females differ in size, color, shape, and appendage development (such as feathers, wattle or appendage, caruncles, beard, and spurs). On the other hand, sexual dimorphism can also be manifested by scent or courtship vocalizations, behavior, and cognition (12). Recent advances in poultry genetics have made it possible to obtain, based on the crosses of given parental strains, offspring showing specific phenotypic traits that make both sexes distinguishable in the early stages of life (13). Genes that modify feather growth have also been described and reported to permit early sex determination (14). However, its implementation in breeding programs was discarded due to a negative impact on the production traits (15). More recent technologies have developed new tools, in which algorithm wing edge detection is used. For this, computational imaging of external wing feathers growth is employed (16). Again, these methods may be difficult to implement in local poultry populations, as morphological and phaneroptic traits may broadly vary across different breeds and varieties.

In avian species, sexual dimorphism is caused by several secondary phenotypical traits that can be recognizable even in the laid egg until the 1-day-old poult (5). Several sex-influenced phenotypical traits in these early stages have been reported, including egg size (17, 18), the opacity of the eggshell (19), feather color, morphology and distribution (20), appendicular skeleton dimensions, focusing on tarsus-metatarsus length (21), head length and size (22, 23), tail inclination (24), and the behavioral performance of the individuals (22, 25).

Considering the aforementioned premises, this study aims to establish which method combination may offer the most efficient and accurate method to determine sex at the early stages of life across the three plumage varieties of the Andalusian turkey breed. This information will be processed to tailor specific non-invasive sexing methods for poult from local turkey populations. The identification of the proportions of individuals belonging to each sex when working with endangered populations can contribute to the improvement and progress of the genetic management tasks carried out in these genotypes. Thus, the tool developed in this study can be a complement to the more commonly used techniques, which have been widely tested on a commercial scale but are sometimes inefficient due to the implicit diversity found in local populations.

## Materials and methods

### Animals and sample size

The present research was conducted in a public hatchery located at the Agropecuary Provincial Center of Diputación of Córdoba (Andalusian, Spain). A total of 18 turkey hens and 3 toms, aged between 12 and 16 months, coming from the base population of the Andalusian turkey breed were reared in three different groups according to plumage color (black: 1M/6F; roan-black: 1M/6F; and bronze-roan: 1M/6F), and were involved in the egg production.

Taking advance of the breeding season (from February to April 2019), eggs were collected daily and stored at 17–18 °C and 70–75 % humidity in incubating platters until their incubation. All eggs were individually numbered, and external egg metrics and eggshell color were determined before incubation.

Eggs intended for incubation were kept for a maximum of 7 days since oviposition. A total of 311 eggs were incubated and divided into seven different incubation periods to ensure sufficient birds are included in the study. An incubator with automatic egg turning (Masalles, M240-I, Barcelona, Spain) was used for 26 days at 37.2 °C and 55 % RH. On the 26th day of incubation, eggs were transferred to a hatchery cabinet (Masalles, 25-N HLC, Barcelona, Spain) maintained at 36.7 °C and 60 % RH until hatching (2 more days). A total of 162 poults hatched, and then were wing-banded and placed in a room with an electric stove to help them regulate their body temperature until performing the sex determination tests.

A random sample of 162 turkey poults (76 black, 58 black-roan, and 28 bronze-roan) was used for sexing. Finally, of the total animals subjected to the sexing tests, we were able to determine the sex of 84 individuals (42 black, 28 black-roan, and 14 bronze-roan). This was due to the fact that some animals died, and others were donated to local farms (as part of an Andalusian turkey breed recovery program) before the sexual dimorphism of the individuals became evident. In the literature, it has been reported that samples of around 100 or even fewer individuals report 95% sexing accuracy in other local poultry species (5, 9, 26). Therefore, of the total of 972 observations that were obtained, only 506 observations were used in the analyzed database, of which the individual sex was confirmed and a complete sexing determination procedure of three appraisers was collected.

Bird management was directed under the European Union Direction regulations (2010/63/EU) as transposed to Spanish Royal Decree-Law 53/2013. This study did not need to be subjected to evaluation by the Ethics Committee of Animal of the University of Córdoba, since it is not part of the legislation for the protection of animals used for scientific purposes.

### External egg metrics and eggshell color

Before incubation, external egg quality was determined in each egg:

- M1 and M2 (major and minor diameters). These measurements were determined using a digital caliper (precision, ±0.01 mm; Electro DH M 60.205, Barcelona, Spain).
- M3 [shape index (SI)]. This index was computed using the following formula (27):

$$SI=\left(\frac{\emptyset M}{\emptyset m}\;\right)\;*100$$

where ØM is the major diameter and Øm is the minor diameter.

If the egg is long and pointed, the individual will be taken as a female, while wide and flat eggs are assigned to males (28). To establish the limits to consider an egg long or flat and wide or broad, the shape index and the median of the diameters were calculated (non-normal distribution, *p* > 0.05), to set over and below the median categories.

- M4 (egg weight). Eggs were weighed individually using an electronic scale (precision ±0.01 g; Cobos, CSB-600C, Barcelona, Spain).
- M5, M6, and M7 (eggshell L^*^, eggshell a^*^, and eggshell b^*^). Eggshell color was assessed using a portable spectrophotometer (CM 700d, Konica Minolta Holdings Inc., Tokyo, Japan), and the results of eggshell color were expressed according to the International Commission on Illumination (CIE) L^*^a^*^b^*^ system color profile.

### Poult morphological appraisal and phaneroptics, behavioral traits, and handling for sexing assignation methods

To carry out the sex determination procedure, each turkey poult was held by the neck during the examination, with the index and middle fingers of the sexer, keeping the poult's head down. Defecation of the animal was caused by applying pressure on the abdominal cavity. Finally, the cloaca was cleaned with a piece of tissue paper (5).

**Figure 1:**
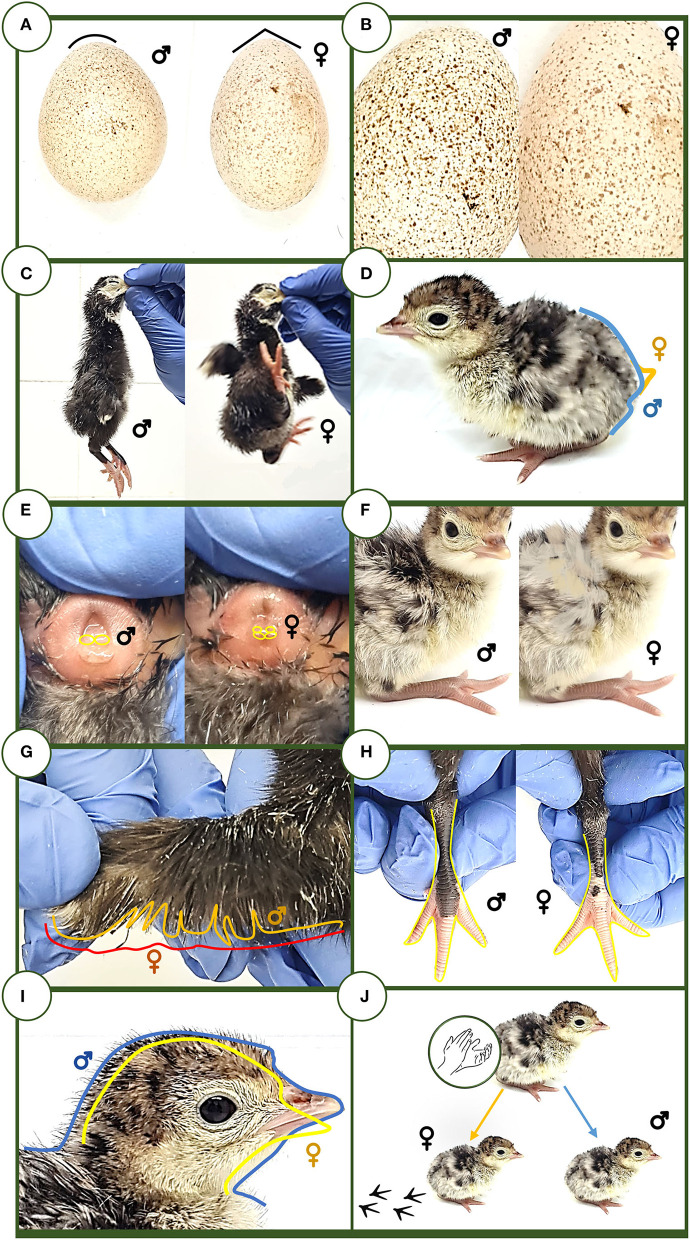
Sex assignment methods. **(A)** Egg length and width test. **(B)** Eggshell color. **(C)** English test. **(D)** Tail inclination. **(E)** Cloaca. **(F)** Side feathers. **(G)** Wing fan. **(H)** Legs. **(I)** Head size and morphology. **(J)** Behavior/coping style.

Various sexing methods based on the poult morphological appraisal and phaneroptics and behavioral traits were performed by three sexers through eight methods, and a dichotomous scale (male or female) was used to classify the animals (5, 29) (Figure 1). The examination tests were performed after hatching, considering it as days 1 and 2 since hatching, and were carried out by three different non-trained evaluators. The different methodologies employed are described as follows:

- M8 (English method). The bird is suspended for 5 s by holding the beak with two fingers, thus analyzing the acquired behavior. If the bird stands still, it is considered male, and if the bird kicks, it will be considered female.
- M9 (Tail inclination method). The turkey poults will be taken as a female when the direction of the tail feathers is toward the ground. However, it will be considered as male if the tail is straight.
- M10 (Japanese method or cloaca examination). As described in the “Introduction” section, vent sexing starts from the basis of the appreciation of morphological visual distinction of genital anatomical structures between sexes in hatched poults by trained experts. Cloaca needs to be externalized by carefully applying pressure with the fingers, and then focusing on the central and ventral parts of it. An individual can be considered a male if it shows a unique outline in the cloaca, or a female if two little bulges are observed.
- M11 (General coloring of down feathers method). This method involves observation of the color pattern displayed by the down feathers on both sides. Individuals displaying a uniform coloration will be considered females, while the poults that exhibit heterogeneous coloring of down feathers will be considered males.
- M12 (Fan-shaped wings and general wing metrics determination). It is based on the growth of primary and secondary reminge feathers of the wings. In this regard, a female is identified when all primary and secondary reminge feathers of the wing show a parallel growth and describe a uniform fan edge. In contrast, a wing that exhibits feathers at a different growth stage, describing an irregular contour, will be considered a characteristic of males.
- M13 (Body size and head morphology method). Males have been described to present proportionally smaller and more rounded heads compared to females, showing a bigger and more angular head shape. To state the limits to consider whether the head of a poult is big or small, the median sizes were computed (the sample was not normally distributed, *p* > 0.05), to set over and below the median categories.
- M14 [Leg length method (from femorotibial joint to the end of the medial phalange)]. Male poults are considered to have long legs when compared to female ones. To state the limits to consider whether the legs are long or short, the average measure of the sizes was computed to set over and below the median categories, since the sample was not normally distributed (*p* > 0.05). The complete leg was considered, and not only the shanks.
- M15 (Behavior/coping styles or slap technique method). Hands are clapped at a prudent distance of 20 cm from the animal. This technique is applied individually for each poult in an isolated place, distant from the rest of the poults. Two different reactions can be observed: freezing (male) and fleeing or attempting to escape (female).

All the methods used in Sections External egg metrics and eggshell color and Poult morphological appraisal and phaneroptics, behavioral traits, and handling for sexing assignation methods of the present work are depicted in Figure 1.

### Sex confirmation

After achieving 25 weeks of age, Andalusian turkey breed females and males exteriorized the secondary sexual characters that enabled the confirmation of the real sex of the individuals.

### Statistical analysis

#### Binary logistic regression

Binary logistic regression was used to fit the statistical model described below. This model represents how the chance of an animal belonging to one of the two possible categories (sexes) may depend on the results for covariates or predictors (sexing methods). In this context, Y was defined as a binary outcome with two categories (sex).

Ordinary least squares (OLS) on a dichotomous dependent variable and binary logistic regression are the two alternatives that can be considered in the case of regressing binary outcomes. OLS is a type of linear least squares method that is used to estimate the unknown parameters in a linear regression model.

Particularly, OLS selects the parameters of a linear function of a set of explanatory variables (sex determination methods) by the principle of least squares, minimizing the sum of the squares of the differences between the observed dependent variable (values of the variable being observed) in the given dataset and those predicted by the linear function of the independent variable.

However, there are three assumptions that must be met prior to running the analyses. First, the error terms need to be heteroskedastic. Thus, the variance of the dependent variable and independent variables must be different, the error terms must not distribute normally, and the predicted probabilities can be > 1 or <0, which can be a problem for subsequent analysis. The “logit” model solves these problems:


$$\begin{array}{l} \text{ln}\left[\text{p}/\left(1-\text{p}\right)\right]=\text{a}+\text{BX} \end{array}$$

where p is the probability that Y for cases equals 1, p (Y=1), 1-p is the probability that Y for cases equals 0, 1–p(Y=1), p/(1-p)” is the odds, and ln[p/1-p] is the log odds, or “logit.”

In logistic regression, we predict Z, not p, because of Z's convenient mathematical properties. Z is a linear function of the predictors, and we can translate that prediction into a probability. The natural log of the odds is called the “logit” = “Z.” Z can be described as follows;


$$\begin{array}{l} \text{Z}=\text{log}\left(\text{p}/1-\text{p}\right)={\text{B}}_{0}+{\text{B}}_{1}\cdot {\text{X}}_{1}+{\text{B}}_{2}\cdot {\text{X}}_{2}+{\text{B}}_{3}\cdot {\text{X}}_{3}\;\ldots \end{array}$$

B's in logistic regression are analogous to b's in OLS, B1 is the average change in Z per one unit increase in X1, controlling for the other predictors, and so on.

The set of independent covariates and categorical predictors (B) consisted of the external egg metrics and eggshell color and sexing methods outcomes using the logistic regression procedure of the Modeling Data Package in XLSTAT Version 2014.5.03 (30). A single model was performed for each of the varieties (black, black-roan, and bronze-roan). The Hosmer–Lemeshow test was used to determine the goodness of fit of the logistic regression model. Essentially, it is chi-square goodness of fit test. When *P* > 0.05 (assuming α = 0.05), we conclude that the logistic regression model is a good fit.

#### Interpreting logistic coefficients

Once significant covariates and predictors have been identified, the sign of Bs will determine the changes in the log odds of the dependent variable, but not changes in the dependent variable (as in OLS). If B for a specific predictor is positive, a unit change in its related x will raise the odds of the event happening, after controlling for the other predictors, while if B is negative, the odds of the event decrease with a unit increase in x.

Exp(B) means “e to the power B” or e^B^. It is called the “odds ratio” (Gr. symbol: Ψ), e is a, mathematical constant used as the “base” for natural logarithms. In logistic regression, eB is the factor by which the odds change when X increases by one unit.


$$\begin{array}{l} \text{New odds}/\text{Old odds}=\text{eB}=\text{odds ratio} \end{array}$$

Odds ratios > 1 indicate a positive relationship between IV and DV (event likely to occur)

Odds ratios <1 indicate a negative relationship between IV and DV (event less likely to occur)

The significance of logistic coefficients is determined by a Wald test. Wald is χ^2^ with 1 df and equals a two-tailed t^2^ with a *p*-value exactly the same.

The knowledge of the distribution of sex yielded the likelihood of the sample. To estimate the B parameters of the model (the coefficients of the linear function), the likelihood function was maximized. As opposed to linear regression, an exact analytical solution does not exist; hence, an iterative algorithm had to be applied.

Maximization of the likelihood function was performed using the Newton–Raphson algorithm with 100 iterations and a convergence level of 0.000001, which are given as default by XLSTAT Version 2014.5.03 (30).

#### Specificity and sensitivity

Sensitivity, true positive rate, or the recall measured the proportion of individuals correctly attributed to sex, and specificity (also called the true negative rate) measured the proportion of individuals incorrectly attributed to sex. These two parameters were computed using the logistic regression procedure of the Modeling Data Package in XLSTAT Version 2014.5.03 (30).

## Results

Table 1 displays the existing correlations across external egg traits. In this case, as the probability of these variables modeling for real sex determination was lower than 0.001 (Tables 1, 2), the variables chosen were concluded to statistically significantly condition and model for real sex determination. Table 3 determined whether the set of variables evaluated in this study may have significantly conditioned (i.e., have been responsible for) real sex determination by comparing the model as it was defined with a simpler model with only one intercept.

**Table 1:** Correlation matrix for external egg characteristics across Andalusian turkey variety pairs.

	**Variable**	**Egg weight**	**Major diameter**	**Minor diameter**	**Shape index**	Eggshell L*	Eggshell a*	Eggshell b*
**Black**	Egg weight	1.0000	0.8810	0.6971	−0.1932	0.0665	−0.0815	−0.1485
	Major diameter		1.0000	0.5155	−0.4339	0.1677	0.0017	−0.1494
	Minor diameter			1.0000	0.3759	0.0023	−0.1902	−0.2408
	Shape index				1.0000	−0.2572	−0.0034	0.0767
	Eggshell L*					1.0000	−0.4750	−0.6504
	Eggshell a*						1.0000	0.5871
	Eggshell b*							1.0000
**Black–roan**	Egg weight	1.0000	0.8042	0.9735	0.0650	−0.1428	−0.1892	0.0767
	Major diameter		1.0000	0.6712	−0.4920	−0.1941	−0.0002	0.2944
	Minor diameter			1.0000	0.2549	−0.0417	−0.2482	−0.0425
	Shape index				1.0000	0.1398	−0.3061	−0.3788
	Eggshell L*					1.0000	0.2621	−0.5066
	Eggshell a*						1.0000	0.3564
	Eggshell b*							1.0000
**Bronze–roan**	Egg weight	1.0000	0.8677	0.5874	−0.5105	0.2303	0.4855	−0.0066
	Major diameter		1.0000	0.6595	−0.6107	0.1625	0.4832	0.2456
	Minor diameter			1.0000	0.1919	0.0869	0.5166	0.3301
	Shape index				1.0000	−0.1142	−0.0915	0.0380
	Eggshell L*					1.0000	0.3305	−0.3803
	Eggshell a*						1.0000	0.0317
	Eggshell b*							1.0000

**Table 2:** Goodness of fit statistics for each Andalusian turkey variety.

	**Black**		**Black–roan**		**Bronze–roan**	
**Statistic**	**Independent**	**Full**	**Independent**	**Full**	**Independent**	**Full**
Observations	252	252	168	168	84	84
Sum of weights	252.0000	252.0000	168.0000	168.0000	84.0000	84.0000
df	251	241	167	157	83	74
−2 Log (Likelihood)	347.0570	323.2748	232.0396	118.2362	114.7286	0.0000
R^2^ (McFadden)	0.0000	0.0685	0.0000	0.4904	0.0000	1.0000
R^2^ (Cox and Snell)	0.0000	0.0901	0.0000	0.4921	0.0000	0.7448
R^2^ (Nagelkerke)	0.0000	0.1204	0.0000	0.6572	0.0000	1.0000
AIC	349.0570	345.2748	234.0396	140.2362	116.7286	20.0000
SBC	352.5864	384.0985	237.1635	174.5998	119.1594	44.3082
Iterations	0	6	0	8	0	24

*Df, degrees of freedom; AIC, Akaike's Information Criterion; SBC/BIC, Schwarz's Bayesian Criterion/Bayesian Information Criterion*.

**Table 3:** Test of the null hypothesis (Black: Y = 0.5476, Roan–black: Y = 0.4643, and Roan–bronze: Y = 0.5714).

	**Black**			**Black–roan**			**Bronze–roan**		
**Statistic**	**df**	**Chi–square**	**Pr > Chi^2^**	**df**	**Chi–square**	**Pr > Chi^2^**	**df**	**Chi–square**	**Pr > Chi^2^**
−2 Log (Likelihood)	10	23.7822	0.0082	10	113.8034	<0.0001	9	114.7286	<0.0001
Score	10	22.9502	0.0109	10	88.5760	<0.0001	9	52.2329	<0.0001
Wald	10	21.2797	0.0192	10	43.1063	<0.0001	9	8.9880 E^−5^	1.0000

Table 2 provides several indicators of the quality of the model (or goodness of fit). These results were equivalent to R^2^ and the analysis of the variance table in linear regression and ANOVA. The most important value was the probability of the chi-square test on the log ratio. This is equivalent to Fisher's F test, and it is used to evaluate whether the variables bring significant information by comparing the model when it is defined with a simpler model with only one constant. In this case, as the probability was lower than 0.0001 (Table 1), we could conclude that data could be significantly modeled by the set of variables chosen.

### Parameter analysis

Table 4 provides details on the model and presents a measure of the effect of the variables considered on the categories of the response variable. There is one intercept for each category of the response variable and one set of coefficients, since the parallel curves hypothesis is supposed to be met.

**Table 4:** Summary of the results for strength of association between the plumage varieties of the Andalusian turkey breed and the ability to succeed or fail when assigning sex for the different methods using the chi–square independence test.

	**Source**	**df**	**Chi–square (Wald)**	**Pr > Wald**	**Chi–square (LR)**	**Pr > LR**
Black	Egg weight	1	0.0114	0.9149	0.0114	0.9149
	Major diameter	1	0.4698	0.4931	0.4708	0.4926
	Minor diameter	1	0.0037	0.9514	0.0037	0.9514
	Shape index	1	0.1405	0.7078	0.1408	0.7075
	Eggshell L*	1	0.0907	0.7632	0.0908	0.7632
	Eggshell a*	1	0.0189	0.8906	0.0189	0.8906
	Eggshell b*	1	1.0740	0.3000	1.0720	0.3005
	English test	1	0.4082	0.5229	0.4078	0.5231
	Down feathers	1	3.8028	0.0512	3.9067	0.0481
	Coping styles	1	7.1448	0.0075	7.2311	0.0072
Black–roan	Egg weight	1	1.5166	0.2181	1.6092	0.2046
	Major diameter	1	0.8368	0.3603	3.8218	0.0506
	Minor diameter	1	1.8873	0.1695	3.3790	0.0660
	Shape index	1	2.7296	0.0985	35.1506	<0.0001
	Eggshell L*	1	7.6134	0.0058	14.3921	0.0001
	Eggshell a*	1	0.0706	0.7905	0.0706	0.7904
	Eggshell b*	1	11.1091	0.0009	15.7778	<0.0001
	English test	1	0.0754	0.7837	0.0753	0.7837
	Down feathers	1	3.9462	0.0470	4.2696	0.0388
	Coping style	1	0.1649	0.6847	0.1653	0.6843
Bronze–roan	Egg weight	1	7.18741E^−5^	0.9932	2811.4050	<0.0001
	Major diameter	1	7.27825E^−5^	0.9932	3460.1907	<0.0001
	Minor diameter	1	7.09201E^−5^	0.9933	2378.8811	<0.0001
	Shape index	1	7.77541E^−6^	0.9978	2234.7065	<0.0001
	Eggshell L*	1	2.66893E^−5^	0.9959	2595.1430	<0.0001
	Eggshell a*	1	5.57253E^−5^	0.9940	2595.1430	<0.0001
	Eggshell b*	1	2.24125E^−9^	1.0000	2595.1430	<0.0001
	English test	1	1.0971E^−8^	0.9999	2595.1430	<0.0001
	Down feathers	1	2.05692E^−8^	0.9999	2595.1430	<0.0001
	Coping style	1	7.18741E^−5^	0.9932	2811.4050	<0.0001

When the regression coefficient for a specific category within a variable was equal to 0.000, this indicated that the said category was taken as the reference to measure the higher or lower repercussions of the subsequent categories in the same variable. The standardized regression coefficient measured the number of times that a certain level or category had a higher (positive standardized coefficient) or lower (negative standardized coefficient) repercussion.

The interpretation of parameters was not immediate. Based on the results in Table 3, it was concluded that the model equation for each variety was as follows:

#### Black variety

Pred(REAL SEX) = 1/[1 + exp(-(-9.01007+0.00635^*^Egg Weight+0.09983^*^Egg Length+0.01051^*^Egg Width+0.03087^*^Shape index-0.00983^*^L^*^-0.02763^*^a^*^-0.04581^*^b^*^-0.19432^*^English method-1+0.65821^*^General coloring of down feathers method-2+0.72086^*^ Behavior/coping styles or slap technique method))]

#### Black-roan variety

Pred(REAL SEX) = 1/[1 + exp(-(-166.45357+0.54722^*^Egg Weight+1.49114^*^Egg length-3.68572^*^Egg Width+2.43531^*^Shape index+0.22455^*^L^*^+0.04886^*^a^*^+0.36373^*^b^*^-0.15040^*^English method-1-1.09496^*^General coloring of down feathers method-1-0.19976^*^ Behavior/coping styles or slap technique method))]

#### Bronze-roan variety

Pred(REAL SEX) = 1 / [1 + exp(-(-42494-233.76746^*^Egg Weight+337.60584^*^Egg length o+822.15956^*^Egg Width+6.06954^*^L^*^-20.84484^*^a^*^-13.93728^*^b^*^+0.21860^*^English method-1-0.85095^*^General coloring of down feathers method-1+0.68119^*^ Behavior/coping styles or slap technique method))]

#### Specificity and sensitivity

Specificity values were 49.12, 93.33, and 100%, while sensitivity values were 74.64, 91.03, and 100%, which translated into the accuracy of 63.10, 92.26, and 100% in black, black–roan, and bronze-roan poults, respectively. A detailed report of the classification table for the estimation sample used to compute the aforementioned parameters is presented in Table 5.

**Table 5:** Classification table for the estimated sexes according to different plumage varieties.

	**From\To**	**Male**	**Female**	**Total**	**% correct**
Black	Male	56	58	114	49.12%
	Female	35	103	138	74.64%
	Total	91	161	252	63.10%
Black–roan	Male	84	6	90	93.33%
	Female	7	71	78	91.03%
	Total	91	77	168	92.26%
Bronze–roan	Male	36	0	36	100.00%
	Female	0	48	48	100.00%
	Total	36	48	84	100.00%

## Discussion

High variability in the ability of the different methods used to predict sex across the different plumage varieties was found. However, the combination of external characteristics of egg, the coloring of down feathers, and behavioral techniques (“English method” and slap technique) reported the best sexing performance with 63.10, 92.26, and 100 % of individuals being correctly classified as black, black-roan, and bronze-roan varieties, respectively (Table 5).

Our results suggest that larger turkey eggs, and hence heavier turkey eggs, are more likely to develop into black and bronze-roan female poults. Literature references have reported a significant relationship between egg metrics and poult sex determination in hens (5, 31). In line with these results, (32) suggested that male turkey poults display higher weights at hatching, as a result of the smaller difference existing between male poult weight and egg preincubation weight than in females. This finding has also been reported for the eggs of other species, such as those of the white-crowned sparrow. In this particular case, the male-containing eggs were larger than the eggs containing females (17) would ascribe this early live sexual dimorphism finding to an adaptive mechanism background in the species (17). This would also be supported by the findings in our study that although male-containing eggs were slightly lighter than the ones containing females in the previously named plumage varieties, no influence of egg size on the adult weight of bird has been reported (33).

Although black Andalusian eggs were larger than those laid by the rest of the plumage varieties, greater difficulties were encountered during the sexing of black Andalusian turkey poults based on the external characteristics of egg. This translates into a disadvantage at the time of sexing, considering the external appearance. Such difficulties may rely on the lower existing variability across eggs of this variety. Indeed, a low genetic variability was reported by (34) in the black plumage variety of the Spanish turkey population. These authors described low values for the number of nucleotides and haplotypes estimated in this population, which is indicative of populations originating from a small number of founders (35). In contrast, high variability in the products of roan varieties may evidence potential traces of hybridization with other nearby Spanish breeds, such as the Oscense and the Minorcan Gall D'Indis turkey breeds, with which Andalusian roans share a similar plumage pattern (7, 36).

Regarding eggshell color, being the descendants of reptiles, ancestral birds were thought to have laid white eggs at first (37). Eggshell pigmentation may have appeared as a mechanism to hide the nest from antipredators, prevent parasitic infestations, or protect the embryo from light-filtering harmful irradiations (38). Furthermore, some species have developed different eggshell pigmentations as a response to other features, such as reinforcement mechanisms for weak shell structure (39), as cooling mechanisms, due to protoporphyrin's ability to reflect infrared light (40), or by the hen to act as a sexual decoy for mating (41).

While eggs containing male poults displayed significantly intenser pigmented eggshells, the color of female-containing eggs was less intense and brighter in the Andalusian turkey breed due to lower pigment depositions. These results are supported by (42), who reported a strong significant association between shell pigmentation intensity and increased male hatching numbers in the barn swallow species. These authors suggested that visual cues about brood sex ratio before egg hatching may let parents prepare for provisioning a highly energy-demanding male-biased brood. Indeed, (43) suggested that turkey hens could adjust the water vapor conductance of eggshells by manipulating carcass porosity, and proposed that secretor cells could adjust the carcass pore number to match embryo metabolism. This maternal ability to influence the egg functional characteristics of turkey, probably mediated by thyroid or iodine metabolism, could affect shell pigment deposition as well, as a response to early sex dimorphism properties in the egg.

The possibility of a hormonal basis in the correlation between sex ratio and egg color could be presumed. However, the mechanism of eggshell color deposition mediated by eggshell glands remains unclear. Contextually, although no influence of blood estrogen or ovulation mechanisms has been determined (44), higher progesterone blood levels prior to ovulation have been proved to influence the accumulation of colored substances in the shell gland, thus in the shell that will eventually be deposited (45), *via* their implication in the activation of the δ-aminolevulinic acid synthetase (46). However, references that either contrast (47) or support our results can be found in the literature, which may provide pieces of evidence of a multifactorial nature for the aforementioned correlation.

According to the Trivers–Willard hypothesis, hens in good metabolic conditions could bias the sex ratio of their progeny toward males, while hens exhibiting poor conditions tend to bias toward females (48–50). In line with this finding, females in good body condition maintain eggshell color to limit visible changes and conceal their eggs in anti-predator behavior. Nevertheless, food-restricted females in lower body conditions modify biliverdin and protoporphyrin concentrations in the eggshell (51). This reinforces the results obtained in the present study, since the ratio of different sexes correlates with the shell color. After the turkey poults are hatched, the present research not only reports acceptable results for the down feather's color method, especially for the black and black-roan patterns, but also allows for a rather efficient early identification of females (better fit). Although this method had been successfully used before for sex determination in hybrid and local fowl strains (13, 52, 53), our study constitutes the first to report its application in the early sex determination of turkeys.

Feather color is strongly influenced by the endocrine system, with thyroid hormone activity being considered one of the most highly conditioning elements of the system (54). Parallelly, pituitary hormones like the α-melanocyte-stimulating hormone (a-MSH), follicle-stimulating hormone (FSH), and luteinizing hormone (LH) are also involved in the plumage coloring process (55), but are thought to be less related to sexual dimorphism and chick feather pigmentation. Sex hormones also influence plumage pigmentation, particularly in terms of sexual dimorphic color pattern, probably acting at the level of melanoblast differentiation (56). Contextually, (54) described that although the expression of feather color is mostly influenced by genetics, estrogen or testosterone levels produce alterations in the plumage pattern of the embryo. In this regard, estrogen has been described to have a high impact on feather color in Brown Leghorn's birds (5). This finding was supported by (52) who was able to differentiate male and castrated female chicks from the New Hampshire x Light Sussex cross with a high success rate, suggesting that early endocrine sexual dimorphism may determine down feather color differences across sexes.

The bronze-roan variety did not report satisfactory results when the down feather's color technique was used. In this way, differences in sexual dimorphism patterns across different genotypes of a single breed population were suggested (5). The most extended feather varieties of Andalusian turkey are black and black-roan, which are originally presented in the ancestral domesticated turkey in Mexico (57). The presence of bronze-roan plumage in the Andalusian breed population may derive from the hypothetical hybridization of individuals belonging to this breed with other similar breeds, which may have translated into the interferences impeding the efficiency of the method.

Although less frequently approached, behavior-based sexing methods, which have barely been included in scientific reports, have reported scarce but interesting results. This gap of knowledge is even larger in turkeys, a species for which worldwide animal production integration is relatively recent. The behavior of this species is comparable to that of other birds that had been domesticated earlier in history (58). The first reference to the scientific application of the “English method” (or “inversion test”) dates back to the past century in Argentina and reported nearly 70% accuracy in the sex determination of hen chicks (29, 59). These results and those in the present article are in line with those reported by (5), who confirmed the significant accuracy of the method for chick sexing in the Utrerana hen breed, a local breed from Spain.

Despite the fact that differences in the reaction to acoustic stimuli between the sexes have been thoroughly studied in chicks (60–67), the behavior/coping style or “slap” technique has scarcely been reported as a sex discriminant technique. It was only (5) who evaluated its applicability in a chick sex-determining study. In line with these results, the “slap technique” reports significant results supporting its feasible applicability for sex determination in domesticated *Meleagris gallopavo* which had never been described in turkey species prior to this study.

The influence of sex on chick behavior has been a widely studied topic during the second half of the 20th century (62, 63, 67, 68). Chick fear was known to inhibit general activity, and scared animals were described to perform both low activity and peeps (62). When 7-day-old chicks were submitted to new stimuli in an ‘open field' test, males were less active and displayed rather fearful responses, displaying freezing, sitting, lying, and eye-enclosure patterns more frequently than females (68).

Chick behavior studies have also focused on isolated animals. In this case, chicks react to loud noises, such as the ring of a bell, and females were much more reactive than males, displaying higher walking and peeping activities and lower freezing, sitting, and eye-enclosure responses (64). Thus, the “English method” can be analogous to this open-field reaction test research made on chicks. The results of the present study showed a lack of activity in males and a higher response when exposed to a new environment, like the hand of the observer, in females. In the “slap technique,” similar results were obtained. Males showed a significant decrease in walking and peeping activity, and thus a significantly decreased reactivity when compared to females. This finding is in contrast to the outcomes of previous research, as males tended to experience increased physiological fear responses, which were reduced after medical tranquilization to the same fear levels displayed by females (64).

Alternative theories propose that higher rates of activity in females (ambulation and distress calls), when compared to males, might not be ascribed to lower fear reactions but to a stronger need to reinstate social contact with conspecifics (69). In addition, male nestlings have shown higher exploration of unfamiliar objects than females when it is required to separate them from their partners, reinforcing less social attachment behavior among males (70). These different responses across chick sexes reflect adult sexual behavior and social organization (71). This way, newly hatched Japanese quail females displayed fear reactions less frequently when a male chick was present (72). This behavior relates to the adult social organization of a certain avian species, where a single male guards a small female group. In this regard, Andalusian turkey females show high social attachment due to their flock idiosyncracies, while males may display lower social needs owing to their solitary nature (73).

The aforementioned sex-related chick responses might be the consequence of early endocrine modulation of post-hatching sexual dimorphism behavior. Contextually, the presence of first steroid hormones in the egg has a maternal origin and plays an important role in the offspring's sex establishment, since they influence post-hatched chick behavior (74). Although the endocrine system is not matured yet, the hormonal synthesis in embryos begins during the egg developmental stage (75, 76). Indeed, the establishment of the hypothalamic–hypophyseal–adrenal axis is known to take place during days 17 and 18 of early development in turkeys, and hormonal activity increases during hatching (77). Hence, differences in the embryonal hormone profiles between sexes could suggest that endocrine sexual dimorphism might influence gallinaceous chicks right from the egg stage.

The aforementioned fact particularly concerned sexual hormones. On the one hand, testosterone has been reported to be the first hormone that is present equally in the plasma of the embryos of both sexes until day 7 of incubation, although a significant increase in the testosterone levels in males is observed (78). On the other hand, (79) described that the embryo ovary produces higher levels of estrogen than the testes during egg development. This produces a higher estrogen/androgen ratio in females, which was suggested to determine the reproductive behavior of adult Japanese quails. Additionally, steroids have proved their influence on both hormone receptors' tissular density and hormone-secreting cell distribution during early development and have direct implications in showing distinct sensitivity to hormones in adulthood (80).

Apart from its influence on physiological fear responses, the influence of hormones on anatomical neural development explains a female's greater reactions and activity (5) explained that a chick's sexual dimorphism behavior may be a direct consequence of the impact of different steroids on the development of visual vias' lateralization. Neural and behavioral lateralization has been reported to play a fundamental role in brain organization, where androgens, especially testosterone, are involved (81). In this sense, several functions are lateralized in avian species (82), and two *vias* are described. First, the ‘right-eye system' is specialized to see large distant objects, whereas the ‘left-eye system' is skilled to analyze the changes in special relations and positions (70) suggested that the specialization of the left-eye system is lower in female chicks than in male ones. This particularly lower space sight could explain the female's particular closeness to the hen and its relatives, and therefore their higher partnership needs described above. When considering the early feeding rates of female chicks after hatching and their higher willingness to eat novel colored food (71), greater development in the right-eye system of female chicks is suggested (83). This phenomenon could support the major reactiveness of females to fear-generating stimuli, which can be attributed to the right-eye system's implication in fixing large and distant objects, similar to the human observer (70).

In the present research, the ‘slap test' not only performed efficiently in black-feathered poults, but this method was also seven times more significant than the other techniques for this plumage. The effects of plumage color on behavior have been extensively studied with contradictory results. Indeed, while in hens, white-feathered individuals have been suggested to be more aggressive than black and gray ones (84, 85). In an indigenous turkey breed, more aggressive behavior has been reported in black- and lavender-feathered individuals when compared to white individuals (86). In line with these results, as aggression can frequently be initiated by a fear-producing stimulus, (87) described white-feathered turkeys may be less fearful than bronze birds.

The basis for this observation may stem from the fact that a significant relationship has been reported between skin pigmentation and certain conduct patterns in many species, such as Norway rats, lions, and wild foxes (88–90). The link between different physiological mechanisms and dark feather patterns has been described to be the result of broad pleiotropic effects of the gene network encoding melanin synthesis or its transport and deposition (42, 91). Genes controlling the deposition of plumage pigments, such as the agouti signaling protein gene, melanocortin-1 gene, or the tyrosine gene, have been proposed to also affect the hormonal status and influence sexual behavior or aggressiveness (92). Thus, darker animals may tend to be more aggressive, possibly due to a higher release of self-stimulating pheromones and a greater secretion of exocrine glands that melanocortins promote (86).

Due to plumage color selection during domestication and its well-studied relationship with behavior (91), higher primitive behavioral gene preservation in black-feathered turkeys should be considered. At this concern, behavior has been closely related to animal domestication, being directly and indirectly modified by humans, since individuals that show better tolerance to human presence also showed the highest production (25, 93, 94). This can be attributed to a lower hypothalamic–hypophyseal–adrenal axis reactivity, a consequence of genetic modifications that selection for docility achieved during domestication (25). For instance, in an open-field test, black-feathered poults displayed greater reactiveness to fear (86). It has been reported that black plumage performed fear-avoidance behaviors (escaping, jumping, and flight) more frequently than lighter-colored individuals.

The effect of early selection practices along with the domestication process of turkeys may be more relevant in the Andalusian turkey breed. The Andalusian turkey breed is a very rudimentary population that has barely been submitted to selection or improvement. This population has often been described as a living representation of the first turkeys that arrived in Europe in the 16th century, and before that, these birds had only accomplished 1,300 years of domestication (10), while comparatively, the chicken species may have probably been domesticated for 4,700 years by that time (95, 96). Therefore, black-feathered Andalusian turkeys could present a greater degree of relatedness to wild ancestors than the individuals presenting one of the remaining plumage patterns.

Bertin and Richard-Yris (97) reported that despite thousands of years of domestication, the free-range-reared domestic animals showed behaviors that still closely resembled those of their wild ancestors. Contextually, the increased frequencies of sex-biased fear responses to strong human stimuli may be the reason why statistically significant reliability in the “slap test” was only reached in black-feathered poults. This hypothesis is supported by previous research in which a non-selected fowl genotype shared more behavioral patterns with a wild ancestor than a highly selected strain (98).

## Conclusion

Conclusively, the combination of egg external characteristics, down feather coloring, and two behavioral techniques (“English method” and slap technique) allows effective sexing in newly hatched poults belonging to the Andalusian turkey breed, chiefly for the two roan varieties (black-roan and bronze-roan). Sexual dimorphism is not very evident in egg size, since egg dimensions do not influence adult weight in turkeys. Early sexual dimorphism is significant when eggshell color is considered, since female-containing eggs were less intensely colored and brighter due to a lower pigment deposition. Color differences of the bronze-roan variety with the predominant black-based colors render this method significantly invalid for sex determination in this plumage pattern. Behavioral techniques like the “English method” and the “slap test” presented high discriminatory power. In any case, the development of this battery of tests, their high predictive potential, and the ease of implementation in non-industrialized farms allows a reliable determination of sexual relationship in a population due to their low economic cost of implementation and the relative improvement in efficiency at data collection, which deems this tool a time and resource-economic alternative to other methods.

## Data availability statement

The original contributions presented in the study are included in the article/Supplementary material, further inquiries can be directed to the corresponding authors.

## Ethics statement

Ethical review and approval was not required for the animal study because Bird management was directed under the European Union Direction regulations (2010/63/EU) as transposed to Spanish Royal Decree-Law 53/2013. This study did not need to be subjected to evaluation by the Ethics Committee of Animal of the University of Córdoba, since it is not part of the legislation for the protection of animals used for scientific purposes.

## Author contributions

JS, FN, and AG: conceptualization and original draft preparation. FN, AG, and AA: methodology and data curation. FN and AA: software and visualization. JL, JD, and MC: validation and formal analysis. JS, FN, AG, and AA: investigation. JD and MC: resources and funding acquisition. MC: project administration. FN and JL: reviewing and editing. FN: supervision. All authors have read and agreed to the published version of the manuscript.

## Funding

This research was funded by FEDER project P20_00893.

## Conflict of interest

The authors declare that the research was conducted in the absence of any commercial or financial relationships that could be construed as a potential conflict of interest.

## Publisher's note

All claims expressed in this article are solely those of the authors and do not necessarily represent those of their affiliated organizations, or those of the publisher, the editors and the reviewers. Any product that may be evaluated in this article, or claim that may be made by its manufacturer, is not guaranteed or endorsed by the publisher.

## References

[1] Clark D Nestor K Velleman S. Continual selection for increased 16 wk body weight on Turkey growth and meat quality: 50 generation update. J Appl Poult Res. (2019) 28:658–68. 10.3382/japr/pfz017

[2] Jahan B Ashraf A Rahman M Molla M Chowdhury S Megwalu F. Rearing of high yielding turkey poults: problems and future prospects in Bangladesh: a review. J Biotechnol Biomed Eng. (2018) 1:1008.

[3] Aviagen. Management Guidelines for Breeding Turkeys. Tattenhall: Aviagen Turkeys Limited (2019). Available online at: http://www.aviagenturkeys.com/uploads/2019/04/10/BR28_V2.1_Management%20Guidelines%20for%20Breeding%20Turkeys_UK.pdf

[4] Steiner G Bartels T Krautwald-Junghanns ME Boos A Koch E. Sexing of turkey poults by Fourier transform infrared spectroscopy. Anal Bioanal Chem. (2010) 396:465–70. 10.1007/s00216-009-3273-z 19936721

[5] Iglesias Pastrana C Navas González FJ Marín Navas C Arando Arbulu A González Ariza A León Jurado JM. Sexual dimorphism for coping styles complements traditional methods for sex determination in a multivariety endangered hen breed. Animals. (2019) 9:1165. 10.3390/ani9121165 31861237 PMC6941311

[6] Gutiérrez-Reinoso MA Aponte PM Cabezas J Rodriguez-Alvarez L Garcia-Herreros M. Genomic evaluation of primiparous high-producing dairy cows: Inbreeding effects on genotypic and phenotypic production–reproductive traits. Animals. (2020) 10:1704. 10.3390/ani10091704 32967074 PMC7552765

[7] Fernández M Gómez M Delgado J. Adán S, Jiménez. M. Guía de campo de las razas autóctonas españolas. SERGA: Madrid, Spain. (2009). p. 683–4.

[8] González Ariza A Navas González FJ Arando Arbulu A León Jurado JM Barba Capote CJ Camacho Vallejo ME. Non-parametrical canonical analysis of quality-related characteristics of eggs of different varieties of native hens compared to laying lineage. Animals. (2019) 9:153. 10.3390/ani9040153 30970531 PMC6523069

[9] Akter S Das S Apu A Ahmed T Lahiry A Afrin A. Early sex determination of Turkey by observation of differences in body weight between male and female. Progress Agric. (2020) 31:218–26.

[10] Arando A González-Ariza A Lupi T Nogales S León J Navas-González F. Comparison of non-linear models to describe the growth in the Andalusian turkey breed. Ital J Anim Sci. (2021) 20:1156–67. 10.1080/1828051X.2021.1950054

[11] Ralls K Mesnick S. “Sexual dimorphism” in Encyclopedia of marine mammals. Amsterdam, Netherlands: Elsevier (2009). p. 1005–11.

[12] Punzalan D Hosken DJ. Sexual dimorphism: why the sexes are (and are not) different. Curr Biol. (2010) 20:972–3. 10.1016/j.cub.2010.09.067 21093787

[13] Göhler D Fischer B Meissner S. In-ovo sexing of 14-day-old chicken embryos by pattern analysis in hyperspectral images (VIS/NIR spectra): a non-destructive method for layer lines with gender-specific down feather color. Poult Sci. (2017) 96:1–4. 10.3382/ps/pew282 27591278

[14] Vučićević M Stevanović J Vučićević I Pantelić A 0elić N Resanović R. Sex determination in game birds management. In Proceedings of the International symposium, on hunting,≫ Modern aspects of sustainable management of game population. Zemun-Belgrade Serbia. (2012) p. 91–4).

[15] Magothe TM Muhuyi WB. Influence of major genes for crested-head, frizzle-feather and naked-neck on body weights and growth patterns of indigenous chickens reared intensively in Kenya. Trop Anim Health Prod. (2010) 42:173–83. 10.1007/s11250-009-9403-y 19579054

[16] Jones P Shearer S Gates R. Edge extraction algorithm for feather sexing poultry chicks. Trans ASAE. (1991) 34:635–0640. 10.13031/2013.31712

[17] Mead PS Morton ML Fish BE. Sexual dimorphism in egg size and implications regarding facultative manipulation of sex in mountain white-crowned sparrows. Condor. (1987) 89:798–803. 10.2307/1368527

[18] Cordero PJ Griffith SC Aparicio JM Parkin DT. Sexual dimorphism in house sparrow eggs. Behav Ecol Sociobiol. (2000) 48:353–7. 10.1007/s00265000025218814315

[19] Alin K Fujitani S Kashimori A Suzuki T Ogawa Y Kondo N. Non-invasive broiler chick embryo sexing based on opacity value of incubated eggs. Comput Electron Agric. (2019) 158:30–5. 10.1016/j.compag.2019.01.029

[20] Cuervo JJ Møller AP. Phenotypic variation and fluctuating asymmetry in sexually dimorphic feather ornaments in relation to sex and mating system. Biol J Linn Soc. (1999) 68:505–529. 10.1111/j.1095-8312.1999.tb01186.x

[21] Hutt F. Sex dimorphism and variability in the appendicular skeleton of the Leghorn fowl. Poult Sci. (1929) 8:202–18. 10.3382/ps.0080202

[22] Blanckenhorn WU. Behavioral causes and consequences of sexual size dimorphism. Ethology. (2005) 111:977–1016. 10.1111/j.1439-0310.2005.01147.x

[23] Verdiglione R Rizzi C. A morphometrical study on the skull of Padovana chicken. Ital J Anim. Sci. (2018) 2810. 10.1080/1828051X.2017.1412810

[24] Gurusinghe CJ Ehrlich D. Sex-dependent structural asymmetry of the medial habenular nucleus of the chicken brain. Cell Tissue Res. (1985) 240:149–52. 10.1007/BF00217568 3995537

[25] Fallahsharoudi A Kock D Johnsson N Ubhayasekera M Bergquist SKA. Domestication effects on stress induced steroid secretion and adrenal gene expression in chickens. Sci Rep. (2015) 5:1–10. 10.1038/srep15345 26471470 PMC4608001

[26] Homma K Siopes TD Wilson WO Mcfarland LZ. Identification of sex of day-old quail (Coturnix coturnix japonica) by cloacal examination. Poult Sci. (1966) 45:469–72. 10.3382/ps.0450469

[27] González Ariza A Arando Arbulu A Navas González FJ Delgado Bermejo JV Camacho Vallejo ME. Discriminant canonical analysis as a validation tool for multivariety native breed egg commercial quality classification. Foods. (2021) 10:632. 10.3390/foods10030632 33802707 PMC8002516

[28] Kaleta E Redmann T. Approaches to determine the sex prior to and after incubation of chicken eggs and of day-old chicks. Worlds Poult Sci J. (2008) 64:391–9. 10.1017/S0043933908000111

[29] Llanos Company M. Sexaje de pollitos. Hojas Divulgadoras Ministerio de Agricultura Dir Gen Capacit. Agrar. (1963) 1:14.

[30] Addinsoft. XLSTAT Version 5, 03. Paris, France: Addinsoft Corp (2014).

[31] Yilmaz-Dikmen B Dikmen S. A morphometric method of sexing white layer eggs. Braz J Poult Sci. (2013) 15:203–10. 10.1590/S1516-635X2013000300006

[32] Burke W. Sex differences in weight of turkey embryos. Poult Sci. (1994) 73:749–53. 10.3382/ps.0730749 8047516

[33] Scott H Phillips R. Egg size in relation to growth of Narragansett turkeys. Poult Sci. (1936) 15:435–8. 10.3382/ps.0150435

[34] Guan X Silva P Gyenai K Xu J Geng T Smith E. Mitochondrial DNA-based analyses of relatedness among Turkeys, Meleagris gallopavo. Biochem Genet. (2015) 53:29–41. 10.1007/s10528-015-9668-y 25820210

[35] Canales Vergara AM Landi V Delgado Bermejo JV Martínez A Cervantes Acosta P Pons Barro Á. Tracing worldwide turkey genetic diversity using D-loop sequence mitochondrial DNA analysis. Animals. (2019) 9:897. 10.3390/ani9110897 31683884 PMC6912331

[36] Monteagudo L Avellanet R Tejedor M Azón R. Genetic studies in the Oscense Turkey by means of microsatellites. XIV Jornadas sobre Producción Animal, Zaragoza, España. (2011) 485–87.

[37] Kilner R. The evolution of egg colour and patterning in birds. Biol Rev. (2006) 81:383–406. 10.1017/S1464793106007044 16740199

[38] Maurer G Portugal SJ Cassey P. An embryo's eye view of avian eggshell pigmentation. J Avian Biol. (2011) 42:494–504. 10.1111/j.1600-048X.2011.05368.x

[39] Sparks NH. Eggshell pigments–from formation to deposition. Avian Biol Res. (2011) 4:162–7. 10.3184/175815511X13228269481875

[40] Gosler AG Higham JP James Reynolds S. Why are birds' eggs speckled? Ecol. Lett. (2005) 8:1105–13. 10.1111/j.1461-0248.2005.00816.x

[41] Moreno J Osorno JL. Avian egg colour and sexual selection: does eggshell pigmentation reflect female condition and genetic quality? Ecol. Lett. (2003) 6:803–6. 10.1046/j.1461-0248.2003.00505.x

[42] Corti M Romano A Costanzo A Bentz AB Navara KJ Parolini M. Protoporphyrin-based eggshell pigmentation predicts hatching success and offspring sex ratio in the barn swallow. J Avian Biol. (2018) 49:012405. 10.1111/jav.01642

[43] Christensen V Ort J. Influence of diet-mediated maternal thyroid alterations on functional properties of turkey eggs. Poult Sci. (1990) 69:1576–81. 10.3382/ps.0691576 2247421

[44] Soh T Koga O. The effects of sex steroid hormones on the pigment accumulation in the shell gland of Japanese quail. Poult Sci. (1994) 73:179–85. 10.3382/ps.0730179 8165162

[45] Hargitai R Herényi M Török J. Eggshell coloration in relation to male ornamentation, female condition and egg quality in the collared flycatcher Ficedula albicollis. J Avian Biol. (2008) 39, 413–422 10.1111/j.0908-8857.2008.04337.x

[46] Miller LK Kappas A. The effect of progesterone on activities of δ-aminolevulinic acid synthetase and δ-aminolevulinic acid dehydratase in estrogen-primed avian oviduct. Gen Comp Endocrinol. (1974) 22:238–44. 10.1016/0016-6480(74)90114-2 4815163

[47] Correa SM Adkins-Regan E Johnson PA. High progesterone during avian meiosis biases sex ratios toward females. Biol Lett. (2005) 1:215–8. 10.1098/rsbl.2004.0283 17148170 PMC1626207

[48] Trivers RL Willard DE. Natural selection of parental ability to vary the sex ratio of offspring. Science. (1973) 179:90–2. 10.1126/science.179.4068.90 4682135

[49] Pike TW Petrie M. Maternal body condition and plasma hormones affect offspring sex ratio in peafowl. Anim Behav. (2005) 70:745–51. 10.1016/j.anbehav.2004.12.020

[50] Geffroy B Douhard M. The adaptive sex in stressful environments. Trends Ecol. (2019) *E*34 628–40. 10.1016/j.tree.2019.02.012 30952545

[51] Duval C Cassey P Mikšík I Reynolds SJ Spencer KA. Condition-dependent strategies of eggshell pigmentation: an experimental study of Japanese quail (Coturnix coturnix japonica). J Exp Biol. (2013) 216:700–8. 10.1242/jeb.077370 23125343

[52] Groenendijk-Huijbers MM. Sex-linked feathering of female hybrid chick embryos hormonally conditioned. Experientia. (1966) 22:302–3. 10.1007/BF01900462 5957211

[53] Abdellatif M. Sexing on down and feather colour in the Dandarawi Egyptian breed. Br Poult Sci. (2001) 42:327–32. 10.1080/00071660120055287 11469551

[54] Leeson S Walsh T. Feathering in commercial poultry I. Feather growth and composition. Worlds Poult Sci J. (2004) 60:42–51. 10.1079/WPS20033

[55] Höhn E Braun C. Hormonal induction of feather pigmentation in ptarmigan. Auk. (1980) 97:601–7. 10.1093/auk/97.3.601

[56] Inaba M Chuong CM. Avian pigment pattern formation: developmental control of macro-(across the body) and micro-(within a feather) level of pigment patterns. Front Cell Dev Biol. (2020) 8:620 10.3389/fcell.2020.00620 PMC7365947 32754601

[57] Crawford R. Introduction to Europe and diffusion of domesticated turkeys from the America. Arch Zootec. (1992) 41:2.

[58] Huff G Huff W Rath N Donoghue A Anthony N Nestor K. Differential effects of sex and genetics on behavior and stress response of turkeys. Poult Sci. (2007) 86:1294–303. 10.1093/ps/86.7.1294 17575174

[59] Lunn JH. Chick sexing. Am Sci. (1948) 36:280–7. 18915053

[60] Nash RF Gallup GG Mcclure MK. The immobility reaction in leopard frogs (Rana pipiens) as a function of noise-induced fear. Psychon Sci. (1970) 21:155–6. 10.3758/BF03331860

[61] Archer J. Effects of testosterone on immobility responses in the young male chick. Behav Biol. (1973) 8:551–6. 10.1016/S0091-6773(73)80047-1 4705437

[62] Faure J Morozeau F. Etude des liaisons entre comportement en open-field et émotivité chez le jeune poussin. Ann Genet Sel Anim. (1975) 7:197–204. 10.1186/1297-9686-7-2-197 22887511 PMC3316250

[63] Archer J. Testosterone and fear behavior in male chicks. Physiol Behav. (1976) 17:561–4. 10.1016/0031-9384(76)90151-7 1013203

[64] Jones R. Responses of male and female domestic chicks to a startling stimulus and the effects of a tranquilliser. Behav Process. (1980) 5:161–72. 10.1016/0376-6357(80)90063-7 24897720

[65] Suarez SD Gallup GG. Predatory overtones of open-field testing in chickens. Learn Behav. (1981) 9:153–63. 10.3758/BF03197812

[66] Jones RB Mills AD. Estimation of fear in two lines of the domestic chick: correlations between various methods. Behav Process. (1983) 8:243–53. 10.1016/0376-6357(83)90015-3 24923713

[67] Jones RB. The tonic immobility reaction of the domestic fowl: a review. Worlds Poult Sci J. (1986) 42:82–96. 10.1079/WPS1986000817586506

[68] Jones R. Sex and strain differences in the open-field responses of the domestic chick. Appl Anim Ethol. (1977) 3:255–61. 10.1016/0304-3762(77)90006-2

[69] Vallorttgara G Zanforlin M. Open-field behavior of young chicks (Gallus gallus): antipredatory responses, social reinstatement motivation, and gender effects. Anim Learn Behav. (1988) 16:359–62. 10.3758/BF03209088

[70] Workman L Andrew RJ. Simultaneous changes in behaviour and in lateralization during the development of male and female domestic chicks. Anim Behav. (1989) 38:596–605. 10.1016/S0003-3472(89)80004-1

[71] Vallortigara G Cailotto M Zanforlin M. Sex differences in social reinstatement motivation of the domestic chick (Gallus gallus) revealed by runway tests with social and nonsocial reinforcement. J Comp Psychol. (1990) 104:361. 10.1037/0735-7036.104.4.361 2282785

[72] Pittet F Houdelier C Lumineau S. Precocial bird mothers shape sex differences in the behavior of their chicks. J Exp Zool A: Ecol Integr Physiol. (2014) 321:265–75. 10.1002/jez.1858 24616263

[73] Marchewka J Watanabe T Ferrante V Estevez I. Review of the social and environmental factors affecting the behavior and welfare of turkeys (Meleagris gallopavo). Poult Sci. (2013) 92:1467–73. 10.3382/ps.2012-02943 23687141

[74] Eising CM Müller W Dijkstra C Groothuis TG. Maternal androgens in egg yolks: relation with sex, incubation time and embryonic growth. Gen Comp Endocrinol. (2003) 132:241–7. 10.1016/S0016-6480(03)00090-X 12812771

[75] Woods JE Podczaski ES. Androgen synthesis in the gonads of the chick embryo. Gen Comp Endocrinol. (1974) 24:413–23. 10.1016/0016-6480(74)90155-5 4615974

[76] Ottinger MA Vom Saal FS. Impact of environmental endocrine disruptors on sexual differentiation in birds and mammals. Horm Behav. (2002) 325:32. 10.1016/B978-012532104-4/50070-6

[77] Wentworth B Hussein M. Serum corticosterone levels in embryos, newly hatched, and young turkey poults. Poult Sci. (1985) 64:2195–201. 10.3382/ps.0642195 4070148

[78] Woods JE Simpson RM Moore PL. Plasma testosterone levels in the chick embryo. Gen Comp Endocrinol. (1975) 27:543–47 10.1016/0016-6480(75)90076-3 767198

[79] Adkins-Regan E. Hormones and sexual differentiation of avian social behavior. Dev Neurosci. (2009) 31:342–50. 10.1159/000216545 19546571

[80] Badyaev AV. Growing apart: an ontogenetic perspective on the evolution of sexual size dimorphism. Trends Ecol. (2002) E17:369–78. 10.1016/S0169-5347(02)02569-7

[81] Pfannkuche KA Bouma A Groothuis TG. Does testosterone affect lateralization of brain and behaviour? A meta-analysis in humans and other animal species. Trans R Soc Lond, B, Biol. (2009) 364:929–42. 10.1098/rstb.2008.0282 19064349 PMC2666087

[82] Güntürkün O Hoferichter H-H. Neglect after section of a left telencephalotectal tract in pigeons. Behav Brain Res. (1985) 18:1–9 10.1016/0166-4328(85)90164-0 3911979

[83] Mench J Andrew R. Lateralization of a food search task in the domestic chick. Behav Neural Biol. (1986) 46:107–14. 10.1016/S0163-1047(86)90570-4 3767825

[84] Bright A. Plumage colour and feather pecking in laying hens, a chicken perspective? Br. Poult Sci. (2007) 48:253–63. 10.1080/00071660701370483 17578687

[85] Nie C Ban L Ning Z Qu L. Feather colour affects the aggressive behaviour of chickens with the same genotype on the dominant white (I) locus. PLoS ONE. (2019) 14:e0215921. 10.1371/journal.pone.0215921 31048862 PMC6497237

[86] Durosaro SO Iyasere OS Oguntade DO Ilori BM Odubola TA Adewunmi AP. Associations between plumage colour and fear behaviour in young Nigerian indigenous turkeys (Meleagris gallopavo). Appl Anim Behav Sci. (2021) 244:105483. 10.1016/j.applanim.2021.105483

[87] Taskin A Karadavut U Çayan H. Behavioural responses of white and bronze turkeys (Meleagris gallopavo) to tonic immobility, gait score and open field tests in free-range system. J Appl Anim Res. (2018) 46:1253–9. 10.1080/09712119.2018.1495642

[88] Cottle CA Price EO. Effects of the nonagouti pelage-color allele on the behavior of captive wild Norway rats (Rattus norvegicus). J Comp Psychol. (1987) 101:390. 10.1037/0735-7036.101.4.390 3691061

[89] Trut LN. Early canid domestication: the farm-fox experiment. Scientifur. (2000) 24:124–124.

[90] West PM Packer C. Sexual selection, temperature, and the lion's mane. Science. (2002) 297:1339–43. 10.1126/science.1073257 12193785

[91] Karlsson AC Kerje S Andersson L Jensen P. Genotype at the PMEL17 locus affects social and explorative behaviour in chickens. Br Poult Sci. (2010) 51:170–77. 10.1080/00071661003745802 20461577

[92] Ducrest AL Keller L Roulin A. Pleiotropy in the melanocortin system, coloration and behavioural syndromes. Trends Ecol. (2008) E23:502–10. 10.1016/j.tree.2008.06.001 18644658

[93] Schütz KE Kerje S Jacobsson L Forkman B Carlborg Ö Andersson L. Major growth QTLs in fowl are related to fearful behavior: possible genetic links between fear responses and production traits in a red junglefowl × White Leghorn intercross. Behav Genet. (2004) 34:121–30. 10.1023/B:BEGE.0000009481.98336.fc 14739702

[94] Jensen P. Behavior genetics and the domestication of animals. Annu Rev Anim Biosci. (2014) 2:85–104. 10.1146/annurev-animal-022513-114135 25384136

[95] Kanginakudru S Metta M Jakati R Nagaraju J. Genetic evidence from Indian red jungle fowl corroborates multiple domestication of modern day chicken. BMC Evol Biol. (2008) 8:1–14. 10.1186/1471-2148-8-174 18544161 PMC2474866

[96] Thornton EK Emery KF. The uncertain origins of Mesoamerican turkey domestication. J Archaeol Method Theory. (2017) 24:328–51. 10.1007/s10816-015-9269-4

[97] Bertin A Richard-Yris MA. Mothers' fear of human affects the emotional reactivity of young in domestic Japanese quail. Appl Anim Behav Sci. (2004) 89:215–31. 10.1016/j.applanim.2004.06.004

[98] Schuètz KE Jensen P. Effects of resource allocation on behavioural strategies: a comparison of red junglefowl (Gallus gallus) and two domesticated breeds of poultry. Ethology. (2001) 107:753–65. 10.1046/j.1439-0310.2001.00703.x

