# Supplementary material for: Traditional sexing methods and external egg characteristics combination allow highly accurate early sex determination in an endangered native turkey breed
Source: Front Vet Sci. 2022 Aug 15;9:948502. doi: 10.3389/fvets.2022.948502 (PMC9420986; doi:10.3389/fvets.2022.948502)
Supplement: Supplementary file 1 [file Table_1.DOCX]

|  | **Source** | **Value** | **Standard error** | **Wald Chi-Square** | **Pr > Chi²** | **Wald Lower bound (95%)** | **Wald Upper bound (95%)** |
| --- | --- | --- | --- | --- | --- | --- | --- |
| **Black** | Intercept | -9.0101 | 9.1018 | 0.9799 | 0.3222 | -26.8493 | 8.8292 |
|  | Egg weight | 0.0063 | 0.0594 | 0.0114 | 0.9149 | -0.1100 | 0.1227 |
|  | Major diameter | 0.0998 | 0.1456 | 0.4698 | 0.4931 | -0.1856 | 0.3853 |
|  | Minor diameter | 0.0105 | 0.1724 | 0.0037 | 0.9514 | -0.3275 | 0.3485 |
|  | Shape index | 0.0309 | 0.0824 | 0.1405 | 0.7078 | -0.1306 | 0.1923 |
|  | Eggshell L* | -0.0098 | 0.0326 | 0.0907 | 0.7632 | -0.0738 | 0.0541 |
|  | Eggshell a* | -0.0276 | 0.2008 | 0.0189 | 0.8906 | -0.4212 | 0.3659 |
|  | Eggshell b* | -0.0458 | 0.0442 | 1.0740 | 0.3000 | -0.1325 | 0.0408 |
|  | English test | -0.1943 | 0.3042 | 0.4082 | 0.5229 | -0.7905 | 0.4018 |
|  | Down feathers | 0.6582 | 0.3375 | 3.8028 | 0.0512 | -0.0033 | 1.3198 |
|  | Coping styles | 0.7209 | 0.2697 | 7.1448 | 0.0075 | 0.1923 | 1.2494 |
| **Black-roan** | Intercept | -166.4536 | 128.3504 | 1.6819 | 0.1947 | -418.0156 | 85.1085 |
|  | Egg weight | 0.5472 | 0.4444 | 1.5166 | 0.2181 | -0.3237 | 1.4181 |
|  | Major diameter | 1.4911 | 1.6301 | 0.8368 | 0.3603 | -1.7038 | 4.6861 |
|  | Minor diameter | -3.6857 | 2.6829 | 1.8873 | 0.1695 | -8.9441 | 1.5727 |
|  | Shape index | 2.4353 | 1.4740 | 2.7296 | 0.0985 | -0.4538 | 5.3244 |
|  | Eggshell L* | 0.2245 | 0.0814 | 7.6134 | 0.0058 | 0.0650 | 0.3841 |
|  | Eggshell a* | 0.0489 | 0.1839 | 0.0706 | 0.7905 | -0.3117 | 0.4094 |
|  | Eggshell b* | 0.3637 | 0.1091 | 11.1091 | 0.0009 | 0.1498 | 0.5776 |
|  | English test | -0.1504 | 0.5479 | 0.0754 | 0.7837 | -1.2243 | 0.9234 |
|  | Down feathers | -1.0950 | 0.5512 | 3.9462 | 0.0470 | -2.1753 | -0.0146 |
|  | Coping styles | -0.1998 | 0.4919 | 0.1649 | 0.6847 | -1.1639 | 0.7644 |

|  | **Source** | **Value** | **Standard error** | **Wald Chi-Square** | **Pr > Chi²** | **Wald Lower bound (95%)** | **Wald Upper bound (95%)** |
| --- | --- | --- | --- | --- | --- | --- | --- |
| **Bronze-roan** | Egg weight | -990.0385 | 116779.3041 | 0.0001 | 0.9932 | -229873.2688 | 227893.1917 |
|  | Major diameter | 554.7981 | 65031.1650 | 0.0001 | 0.9932 | -126903.9432 | 128013.5393 |
|  | Minor diameter | 790.8336 | 93907.5324 | 0.0001 | 0.9933 | -183264.5477 | 184846.2150 |
|  | Shape index | 0.0000 | 0.0000 | - | - | - | - |
|  | Eggshell L* | 23.3487 | 8373.3832 | 0.0000 | 0.9978 | -16388.1808 | 16434.8782 |
|  | Eggshell a* | -38.3885 | 7430.7386 | 0.0000 | 0.9959 | -14602.3686 | 14525.5916 |
|  | Eggshell b* | -32.3696 | 4336.2103 | 0.0001 | 0.9940 | -8531.1856 | 8466.4465 |
|  | English test | 0.0589 | 1243.2787 | 0.0000 | 1.0000 | -2436.7225 | 2436.8402 |
|  | Down feathers | -0.2264 | 2161.3719 | 0.0000 | 0.9999 | -4236.4375 | 4235.9847 |
|  | Coping styles | 0.1873 | 1305.9596 | 0.0000 | 0.9999 | -2559.4464 | 2559.8210 |
