# Supplementary material for: Traditional sexing methods and external egg characteristics combination allow highly accurate early sex determination in an endangered native turkey breed
Source: Front Vet Sci. 2022 Aug 15;9:948502. doi: 10.3389/fvets.2022.948502 (PMC9420986; doi:10.3389/fvets.2022.948502)
Supplement: Supplementary file 2 [file Table_2.DOCX]

|  | **Source** | **Value** | **Standard error** | **Wald Chi-Square** | **Pr > Chi²** | **Wald Lower bound (95%)** | **Wald Upper bound (95%)** |
| --- | --- | --- | --- | --- | --- | --- | --- |
| **Black** | Egg weight | 0.0213 | 0.1993 | 0.0114 | 0.9149 | -0.3692 | 0.4118 |
|  | Major diameter | 0.1392 | 0.2032 | 0.4698 | 0.4931 | -0.2589 | 0.5374 |
|  | Minor diameter | 0.0098 | 0.1604 | 0.0037 | 0.9514 | -0.3046 | 0.3242 |
|  | Shape index | 0.0494 | 0.1319 | 0.1405 | 0.7078 | -0.2091 | 0.3080 |
|  | Eggshell L* | -0.0314 | 0.1041 | 0.0907 | 0.7632 | -0.2355 | 0.1727 |
|  | Eggshell a* | -0.0137 | 0.0998 | 0.0189 | 0.8906 | -0.2094 | 0.1820 |
|  | Eggshell b* | -0.1182 | 0.1141 | 1.0740 | 0.3000 | -0.3419 | 0.1054 |
|  | English test | -0.0478 | 0.0748 | 0.4082 | 0.5229 | -0.1943 | 0.0988 |
|  | Down feathers | 0.1580 | 0.0810 | 3.8028 | 0.0512 | -0.0008 | 0.3167 |
|  | Coping styles | 0.1987 | 0.0743 | 7.1448 | 0.0075 | 0.0530 | 0.3444 |
| **Black-roan** | Egg weight | 2.2408 | 1.8196 | 1.5166 | 0.2181 | -1.3255 | 5.8070 |
|  | Major diameter | 2.0186 | 2.2067 | 0.8368 | 0.3603 | -2.3065 | 6.3438 |
|  | Minor diameter | -3.2721 | 2.3818 | 1.8873 | 0.1695 | -7.9403 | 1.3962 |
|  | Shape index | 2.6103 | 1.5800 | 2.7296 | 0.0985 | -0.4864 | 5.7070 |
|  | Eggshell L* | 0.9916 | 0.3594 | 7.6134 | 0.0058 | 0.2872 | 1.6959 |
|  | Eggshell a* | 0.0565 | 0.2127 | 0.0706 | 0.7905 | -0.3604 | 0.4735 |
|  | Eggshell b* | 0.9052 | 0.2716 | 11.1091 | 0.0009 | 0.3729 | 1.4375 |
|  | English test | -0.0367 | 0.1338 | 0.0754 | 0.7837 | -0.2989 | 0.2255 |
|  | Down feathers | -0.3018 | 0.1519 | 3.9462 | 0.0470 | -0.5996 | -0.0040 |
|  | Coping styles | -0.0542 | 0.1334 | 0.1649 | 0.6847 | -0.3157 | 0.2073 |

|  | **Source** | **Value** | **Standard error** | **Wald Chi-Square** | **Pr > Chi²** | **Wald Lower bound (95%)** | **Wald Upper bound (95%)** |
| --- | --- | --- | --- | --- | --- | --- | --- |
| **Bronze-roan** | Egg weight | -233.7675 | 27573.8785 | 0.0001 | 0.9932 | -54277.5763 | 53810.0413 |
|  | Major diameter | 337.6058 | 39572.7785 | 0.0001 | 0.9932 | -77223.6147 | 77898.8264 |
|  | Minor diameter | 822.1596 | 97627.3271 | 0.0001 | 0.9933 | -190523.8854 | 192168.2045 |
|  | Shape index | 0.0000 | 0.0000 | - | - | - | - |
|  | Eggshell L* | 6.0695 | 2176.6775 | 0.0000 | 0.9978 | -4260.1400 | 4272.2791 |
|  | Eggshell a* | -20.8448 | 4034.8723 | 0.0000 | 0.9959 | -7929.0492 | 7887.3595 |
|  | Eggshell b* | -13.9373 | 1867.0312 | 0.0001 | 0.9940 | -3673.2512 | 3645.3766 |
|  | English test | 0.2186 | 4617.3729 | 0.0000 | 1.0000 | -9049.6660 | 9050.1032 |
|  | Down feathers | -0.8509 | 8124.1747 | 0.0000 | 0.9999 | -15923.9408 | 15922.2389 |
|  | Coping styles | 0.6812 | 4749.6292 | 0.0000 | 0.9999 | -9308.4210 | 9309.7834 |
